# Longitudinal Associations Among Academic Burnout, Fear of Missing Out, and Smartphone Use Addiction in Chinese University Students: A Two-Wave Study

**DOI:** 10.3390/bs16061034

**Published:** 2026-06-20

**Authors:** Rubin Shi, Ruiqin Xie, Weiyi Xie, Lei Mo

**Affiliations:** 1 Center for Studies of Psychological Application, South China Normal University, Guangzhou 510631, China; shirubin@m.scnu.edu.cn; 2 Key Laboratory of Brain, Cognition and Education Sciences, Ministry of Education, South China Normal University, Guangzhou 510631, China; 3 School of Public Administration, South China Agricultural University, Guangzhou 510642, China; 4 Center for Education and Development of Nanhai District, Foshan 528299, China; xieruiqin@m.scnu.edu.cn

**Keywords:** academic burnout, fear of missing out, smartphone use addiction, cross-lagged panel analysis

## Abstract

Smartphone use addiction and academic burnout represent prevalent phenomena, and existing research indicates a strong positive association between them. However, the longitudinal associations and potential explanatory mechanisms underlying this association remain insufficiently examined. This research explored the reciprocal influences between academic burnout and smartphone use addiction across time, while also examining whether fear of missing out (FoMO) functions as a central mediating mechanism. This research utilized a two-wave longitudinal design, with data collected from participants at two time points separated by a six-month interval. The sample consisted of 893 students from a university in South China. Measures included the Adolescent Student Burnout Inventory, the Fear of Missing Out Scale, and the Mobile Phone Addiction Scale. This research employed an analytical method of cross-lagged panel models with mediating effects. The results demonstrated that smartphone use addiction and academic burnout positively predicted each other over time. Furthermore, FoMO significantly mediated these bidirectional longitudinal associations. These results provide preliminary evidence for bidirectional temporal associations between academic burnout and smartphone use addiction and identify FoMO as one potential mechanism linking the two phenomena over time. These findings offer practical insights for developing targeted intervention strategies to address these interrelated issues among university students.

## 1. Introduction

With the development of information technology, the popularity of smartphones in people’s lives has increased substantially. Although smartphones have facilitated many aspects of daily life, their overuse has led to the emergence of addictive behavior (Mason et al., 2022). Smartphone use addiction is a behavioral dependence characterized by an inability to regulate smartphone use, commonly accompanied by withdrawal symptoms, growing tolerance, and significant impairments in social functioning (Qiu et al., 2025; L. Zhang et al., 2024). Existing research shows that approximately 28.3% of people worldwide experience smartphone use addiction (Xiong et al., 2021). A meta-analysis reveals significantly higher prevalence rates in Asian countries, with China exhibiting the highest rate among 24 nations examined (Olson et al., 2022). Smartphone use addiction is closely linked to several mental health concerns, including anxiety, depression, and sleep disorders (Dou et al., 2024; Feng et al., 2022; L.-L. Yang et al., 2023). University students frequently use smartphones for communication and entertainment. However, due to the increase in free time, the weakening of external supervision and psychological immaturity, smartphone use addiction has become increasingly prevalent among university students (W. Wang et al., 2024). Accordingly, identifying the factors associated with smartphone use addiction and clarifying its underlying mechanisms among university students is essential for informing effective, evidence-based prevention and intervention efforts.

Smartphone use addiction arises from a complex interplay between individual and environmental factors. At the individual level, personality traits, affective states, and cognitive processes play pivotal roles, whereas environmental factors—such as negative life events and interpersonal stressors—further amplify risk (Brand et al., 2019; Tian et al., 2025). Within the university context, academic performance serves as a critical benchmark for evaluating student competence. Consequently, Chinese university students, who face intense competitive pressures and uncertain employment prospects, are particularly susceptible to academic burnout (Z. Liu et al., 2023). Therefore, the present study first examines the influence of academic burnout on smartphone use addiction. Academic burnout refers to a negative physical, psychological, and behavioral state that arises when individuals are exposed to prolonged academic pressure, resulting in diminished learning interest and reduced sense of achievement (L. Yang et al., 2025; Yu et al., 2025). Academic burnout originated from the conceptualization of job burnout and is similarly characterized by three core dimensions: exhaustion, cynicism, and inefficacy (Maslach et al., 2001). When seeking to alleviate negative emotions associated with academic burnout, individuals may overuse their smartphones as an unhealthy coping mechanism (Liou et al., 2022; Z. Wei et al., 2025). Supporting this view, existing research indicates that smartphone use addiction is positively correlated with academic burnout and negatively correlated with academic performance (Samaha & Hawi, 2016; C. Jin et al., 2026; X. Wang et al., 2026). Nevertheless, prior evidence examining this association is overwhelmingly cross-sectional, with longitudinal studies remaining relatively limited. Consequently, the directionality of the association—whether academic burnout predicts smartphone use addiction, or the reverse—remains largely undetermined.

Regarding the etiological mechanisms of smartphone use addiction, the Cognitive-Behavioral Model provides an integrated theoretical framework that categorizes contributing factors into distal and proximal factors (Davis, 2001). Distal factors influence smartphone use addiction by affecting proximal factors. Distal factors include psychopathological factors such as social anxiety and depression. Maladaptive cognition is an important proximal factor that triggers smartphone use addiction. Empirical studies have supported the indirect pathway from distal psychopathology to addiction via these maladaptive cognitions (Stanciu & Calugar, 2022; Tian et al., 2023). Although Davis’s (2001) model does not explicitly locate academic burnout within its distal-proximal framework, accumulating evidence indicates a consistent positive association between academic burnout and symptoms of anxiety and depression (Jiang et al., 2021; Lin et al., 2025; W. Liu et al., 2024). This pattern aligns with conceptualizing academic burnout as a necessary distal vulnerability that may predispose individuals to symptoms of smartphone use addiction. Within this framework, we posit that academic burnout functions as a distal stressor that influences smartphone use addiction both directly and indirectly through maladaptive cognitive processes—specifically, fear of missing out (FoMO)—which operates as a proximal mediating factor (Tian et al., 2025; Wu-Ouyang, 2022; H. Wei et al., 2022).

Fear of Missing Out (FoMO) describes a widespread concern that others are engaging in rewarding or enjoyable experiences without one’s participation, which is frequently conceptualized as a form of social anxiety (Przybylski et al., 2013; Y. Zhang et al., 2025). Essentially, FoMO is characterized by an irrational cognitive preoccupation with others’ activities, accompanied by persistent anxiety (Chai et al., 2018). To alleviate these feelings, individuals often engage in compulsive smartphone use, such as repeatedly checking social media updates. Meta-analytic evidence reveals a moderate and statistically significant association between FoMO and smartphone use addiction (Fioravanti et al., 2021; Y. Zhang et al., 2023). Research suggests that psychopathological factors may be reciprocally associated with addictive behavior, with maladaptive cognitions serving as mediators in these associations (Elhai et al., 2020a; Tian et al., 2025; Y. Wang et al., 2022; C. Liu et al., 2025; Yao et al., 2025; Zhou et al., 2021). Previous studies have consistently shown that academic burnout is closely linked to psychological issues, including depression and anxiety (Lin et al., 2025; Z. Liu et al., 2023). Therefore, we hypothesize that academic burnout is a vulnerability factor for smartphone use addiction, and that FoMO mediates the association between academic burnout and smartphone use addiction. Furthermore, existing research indicates that smartphone use addiction can lead to academic procrastination and FoMO, and FoMO is associated with academic burnout (Han et al., 2025; F. Liu et al., 2022; Y. Zhang et al., 2021). However, the current research on the associations between academic burnout, smartphone use addiction, and FoMO is mostly cross-sectional. In this regard, a longitudinal design was employed to investigate the bidirectional association between academic burnout and smartphone use addiction among university students, and to assess the mediating effect of FoMO.

### 1.1. The Mutual Predictive Association Between Academic Burnout and Smartphone Use Addiction

The Compensatory Internet Use Theory proposes that when basic psychological needs—including the need for competence, autonomy, and relatedness—are unmet in offline contexts, individuals are motivated to seek compensatory gratification through online engagement (Kardefelt-Winther, 2014). For university students, academic performance represents a primary source of perceived competence. Accordingly, academic burnout can be understood as an emotional reaction that emerges when needs for competence are chronically thwarted. When students fail to derive satisfaction from academic pursuits, they may resort to the smartphone to alleviate emotional distress. Furthermore, consistent with the Uses and Gratifications Theory (Katz et al., 1974), when smartphones provide positive experiences such as enjoyment and self-affirmation, these reinforcing effects may promote increasingly frequent and dependent use, eventually culminating in addictive patterns (Ran et al., 2022). Several empirical studies have supported this hypothesized pathway. For example, one study found a positive link between academic burnout and smartphone use addiction among undergraduate students, with anxiety serving as a mediating factor (Hao et al., 2021). Moreover, a meta-analysis indicated that individuals experiencing higher levels of academic burnout tended to show greater severity in smartphone use addiction (S. Li et al., 2023).

Moreover, smartphone use addiction may increase the likelihood of experiencing academic burnout. According to the Cognitive Processing Model of Addiction, individuals addicted to smartphones often form automatic stimulus-response patterns through repeated engagement, leading to attentional biases toward smartphone-related cues (L. Chen et al., 2018; Wiers & Stacy, 2006). Such biases have been shown to significantly predict loss of control over addictive behaviors (Dean et al., 2019; Rinck et al., 2018). Furthermore, Media Dependency Theory posits that the more functions a medium provides, the greater individuals’ dependence on it (Ball-Rokeach & DeFleur, 1976). When people rely too heavily on a medium to fulfill their psychological needs, such dependence may result in harmful consequences. Drawing on these theoretical frameworks, university students with smartphone use addiction—particularly those immersed in virtual environments—are more likely to experience attentional distraction during learning, difficulties in completing tasks on time, and disengagement from academic pursuits (Wilmer et al., 2017; G.-H. Yang et al., 2024). Over time, academic performance may progressively decline, ultimately triggering academic burnout. Empirical studies support this pathway. Smartphone use addiction weakens students’ concentration during learning (Rosen et al., 2013), exacerbates academic procrastination (Geng et al., 2018; F. Liu et al., 2022), reduces academic engagement (Y. Zhang et al., 2018), and leads to a decline in academic performance (Lepp et al., 2014), thereby increasing the risk of academic burnout (He & Xia, 2019). A meta-analysis covering 44 studies further indicates that smartphone use addiction has detrimental effects on academic performance, such that more frequent use during learning is associated with greater decrements in academic achievement (Sunday et al., 2021).

However, most existing research on the association between these two variables has relied on cross-sectional data. This methodological limitation precludes the identification of temporal precedence and reciprocal influences, thereby hindering the development of evidence-based interventions. Consequently, longitudinal investigations are imperative to further elucidate the bidirectional associations and underlying mechanisms between them.

### 1.2. The Bidirectional Mediating Role of FoMO in the Association Between Academic Burnout and Smartphone Use Addiction

From a cognitive-affective-behavioral perspective, FoMO manifests cognitively as a persistent desire to keep up with others’ activities, affectively as apprehension about missed experiences, and behaviorally as compulsive smartphone use (Chai et al., 2018). Essentially, FoMO represents a complex psychological construct encompassing maladaptive cognition, anxious affect, and dysfunctional behavioral patterns. Empirical studies suggest that FoMO, understood as a key maladaptive cognitive tendency, acts as an important proximal factor contributing to addictive smartphone use (Tao et al., 2023; Y. Wang et al., 2022; Wu-Ouyang, 2022). According to Self-Determination Theory (Deci & Ryan, 2000), effective self-regulation relies on the satisfaction of three basic psychological needs: autonomy, competence, and relatedness. When these basic psychological needs are thwarted, self-regulatory processes may become impaired, giving rise to FoMO (Chai et al., 2018). Research indicates that basic psychological need satisfaction is negatively associated with FoMO (Przybylski et al., 2013), whereas need frustration—manifested as insufficient sense of belonging, loneliness, and depressive symptoms—is positively correlated with FoMO (Alt, 2015; Y. Chen et al., 2025; Oberst et al., 2017). To compensate for frustrated psychological needs, individuals frequently turn to smartphone-mediated activities (e.g., watching short videos) as alternative sources of need satisfaction. Przybylski et al. (2013) note that the frustration of basic psychological needs may indirectly drive smartphone use through FoMO, because individuals with unmet needs are more sensitive to “missing out”. Academic burnout, characterized by diminished motivation in response to persistent academic strain, may arise from thwarted competence needs. Existing research indicates that negative emotional states, including anxiety, stress, and diminished academic motivation, predict smartphone use addiction via FoMO (Elhai et al., 2020b; H. Yang et al., 2021; Alt, 2015). Longitudinal findings suggest that loneliness in college students contributes to smartphone use addiction, with FoMO acting as an intermediary mechanism (Y. Wang et al., 2025). Therefore, we hypothesize that FoMO will mediate the association between academic burnout and smartphone use addiction.

Research also suggests a bidirectional association, where smartphone use addiction can intensify academic burnout, with FoMO serving as an important mediating factor. Grounded in the displacement hypothesis (Kraut et al., 1998), excessive smartphone use may displace real-world social activities by consuming the time and psychological resources that would otherwise be allocated to in-person interactions. Consequently, reduced offline interpersonal engagement diminishes opportunities for fulfilling social experiences, leading individuals to perceive themselves as excluded from rewarding real-world social contexts. Over time, this lack of real-world social engagement can contribute to elevated FoMO, as individuals perceive themselves to be excluded from fulfilling social experiences. Thus, smartphone use addiction is hypothesized to function as a significant positive predictor of FoMO. Longitudinal studies suggest that more frequent use of social networking sites leads to increases in FoMO (Buglass et al., 2017; Fang et al., 2026; Zhao, 2024).

In addition, FoMO has been consistently linked to diminished learning motivation and poorer academic performance among students, while also heightening susceptibility to academic burnout. Conservation of Resources Theory posits that individuals possess finite pools of cognitive and temporal resources within a given period (Hobfoll, 2011). However, as a persistent state of anxiety, FoMO can cause students to over-allocate their limited cognitive resources, such as time or attention, to online activities on their mobile phones. As a result, the cognitive resources required for sustained, in-depth learning are gradually consumed. Thus, this misallocation may manifest in effects including reduced learning efficiency, diminished academic performance, and lower academic self-efficacy (Guo et al., 2022). Longitudinal accumulation of resource depletion and academic failures significantly heightens the likelihood of academic burnout (C. Liu et al., 2022; Ye et al., 2023). Taken together, the available evidence suggests that smartphone use addiction may indirectly contribute to heightened academic burnout via the exacerbation of FoMO. Existing studies indicate that negative affect may contribute to smartphone use addiction indirectly through heightened FoMO (Elhai et al., 2020b; C. Liu et al., 2025). However, existing research examining the associations among smartphone use addiction, academic burnout, and FoMO has predominantly relied on cross-sectional designs focusing on pairwise associations. Longitudinal investigations into whether academic burnout prospectively influences smartphone use addiction, and whether FoMO serves as an underlying mediating mechanism in this association, remain relatively scarce. This methodological limitation constrains the development of evidence-based intervention strategies targeting these interrelated issues.

Based on the theoretical framework and prior empirical findings, this study proposes the following hypotheses:

**H1.**  *Academic burnout and smartphone use addiction will positively and reciprocally predict each other over time.*

**H2.**  *FoMO is expected to serve as a bidirectional mediator between academic burnout and smartphone use addiction.*

## 2. Materials and Methods

### 2.1. Participants

Through convenience sampling, students from 40 classes at a university in Guangdong Province, southern China, were recruited for the study. The chosen institution is a comprehensive “Double First-Class” university with multiple disciplines, where students face considerable academic competition pressure. The first wave of data collection (T1) was conducted in November 2024, and yielded 992 participants. Six months later (May 2025), the second wave (T2) was completed. At both waves, participants provided their university student ID number, which was used as a unique identifier to match individual responses across time points. Attrition was primarily due to transferring to other majors or voluntary withdrawal. The final sample comprised 893 university students, including 687 females (76.9%) and 206 males (23.1%). The participants’ average age was 19.93 years (SD = 1.24). At T1, participants were distributed across academic years as follows: 432 freshmen (48.4%) and 461 juniors (51.6%). Regarding family residence, 116 participants (13.0%) were from rural areas, 119 (13.3%) from county towns, 258 (28.9%) from ordinary prefectural-level cities, 208 (23.3%) from developed prefectural-level cities, 27 (3.0%) from ordinary provincial capitals, and 165 (18.5%) from first-tier cities. Monthly income (in CNY) was distributed as follows: ≤2000 (1.3%), 2001–4000 (2.6%), 4001–6000 (9.7%), 6001–8000 (12.3%), 8001–10,000 (20.2%), 10,001–20,000 (34.6%), and >20,000 (19.3%).

### 2.2. Procedure

Data were collected through paper-and-pencil surveys that trained research assistants administered in classrooms. The survey required approximately 15 min to complete. All participants provided written informed consent and were compensated modestly for their participation.

### 2.3. Measures

#### 2.3.1. Adolescent Student Burnout Inventory

Academic burnout was assessed using the Adolescent Student Burnout Inventory (Y. Wu et al., 2010). Although this scale was originally developed for adolescent students, subsequent research has validated its factorial structure and psychometric properties among Chinese university students (Luo et al., 2018). Confirmatory factor analyses in the present study further supported the three-factor structure at both time points. At T1, the model showed acceptable fit to the data: *χ*^2^(98) = 392.435, *p* < 0.001, *χ*^2^/*df* = 4.00; RMSEA = 0.058 (90% CI [0.052, 0.064]); TLI = 0.900; CFI = 0.919; SRMR = 0.058. At T2, the model demonstrated comparable fit: *χ*^2^(98) = 388.191, *p* < 0.001, *χ*^2^/*df* = 3.96; RMSEA = 0.058 (90% CI [0.052, 0.064]); TLI = 0.903; CFI = 0.921; SRMR = 0.062. The 16-item scale includes items such as “I have felt very empty inside recently and do not know what to do” and “I am so bad at studying that I really want to give up.” Participants responded using a 5-point Likert scale ranging from 1 (strongly disagree) to 5 (strongly agree), with higher total scores reflecting higher levels of academic burnout. The measure demonstrated good internal consistency, with Cronbach’s α values of 0.85 at T1 and 0.86 at T2.

#### 2.3.2. Fear of Missing Out Scale

FoMO was measured using the Fear of Missing Out Scale (Przybylski et al., 2013) in its Chinese adaptation (Q. Li et al., 2019). The 8-item scale includes items such as “I am afraid that others have more exciting experiences and gains than I do” and “When traveling, I still closely follow my friends’ latest updates.” Participants rated each item using a 5-point Likert scale ranging from 1 (strongly disagree) to 5 (strongly agree). Total scores were obtained by summing the responses, with higher scores indicating greater FoMO. Cronbach’s α was 0.82 at both T1 and T2.

#### 2.3.3. Mobile Phone Addiction Scale

Smartphone use addiction was measured using the Mobile Phone Addiction Scale (Leung, 2008). The 17-item scale includes items such as “I find it difficult to switch off my mobile phone” and “My productivity has decreased as a direct result of the time I spend on the mobile phone.” Participants rated items on a 5-point Likert scale ranging from 1 (never) to 5 (always), with higher total scores reflecting more severe smartphone use addiction. Cronbach’s α was 0.84 at T1 and 0.90 at T2.

### 2.4. Data Analysis

Based on existing literature, gender, age, family residence, and income are associated with the key variables under investigation (Sunday et al., 2021). Accordingly, these variables were controlled for in the subsequent analyses. Descriptive statistics, correlation analyses, and paired samples t-tests were performed using SPSS 27.0. Cross-lagged panel modeling and mediation analysis were conducted using Mplus 8.3. The fit of the model was evaluated using *χ*^2^/*df*, CFI, TLI, RMSEA, and SRMR. Based on previous research, the following thresholds were adopted to indicate good model fit: *χ*^2^/*df* ≤ 5, CFI > 0.95, TLI > 0.95, and RMSEA < 0.05 (Browne & Cudeck, 1993).

### 2.5. Common Method Bias Testing

To evaluate the potential presence of common method bias, we performed Harman’s single-factor test as recommended by Podsakoff et al. (2003). An exploratory factor analysis (EFA) was carried out, incorporating all items across the three measurement scales. At T1, ten factors emerged with eigenvalues above 1.0, with the largest unrotated factor explaining 19.46% of the variance. At T2, eight factors had eigenvalues greater than 1, with the first unrotated factor accounting for 23.86% of the total variance. At both time points, the first unrotated factor explained less than 40% of the total variance, indicating that common method bias was not a serious concern in this study.

## 3. Results

### 3.1. Descriptive Statistics and Correlation Analysis

Table 1 presents the means (M), standard deviations (SD), and Pearson correlation coefficients for all core variables across the two measurement points (T1 and T2). Gender showed a positive correlation with T1 smartphone use addiction, T2 smartphone use addiction, and T2 FoMO. Age was negatively correlated with T1 FoMO, T2 academic burnout, and T2 FoMO. Family residence was not significantly correlated with any core variables. Income was positively correlated with T2 FoMO. All core variables (T1 academic burnout, T1 FoMO, T1 smartphone use addiction, T2 academic burnout, T2 FoMO, and T2 smartphone use addiction) were significantly and positively intercorrelated.

The paired-samples t-test revealed a significant increase in smartphone use addiction levels from T1 to T2 (*t* = 16.57, *SE* = 0.32, *p* < 0.001). T2 academic burnout was significantly lower than T1 academic burnout (*t* = −6.72, *SE* = 0.24, *p* < 0.001); and T2 FoMO was significantly higher than T1 FoMO (*t* = 28.57, *SE* = 0.19, *p* < 0.001).

### 3.2. Longitudinal Measurement Invariance

To ensure that observed changes across the six-month interval reflect true score differences rather than measurement artifacts, we tested longitudinal measurement invariance for all three constructs using Mplus 8.3. A hierarchy of nested models was examined: configural invariance (M1), weak invariance (M2), strong invariance (M3), and partial strong invariance (M4). Model fit was evaluated using CFI, TLI, RMSEA, and SRMR. Invariance was supported if |ΔCFI| ≤ 0.010 and |ΔRMSEA| ≤ 0.015 (F. F. Chen, 2007).

As shown in Table 2, full strong invariance was established for the Adolescent Student Burnout Inventory and the Fear of Missing Out Scale. For the Mobile Phone Addiction Scale, weak invariance was supported (M2 vs. M1: ΔCFI = −0.005, ΔRMSEA = 0.001), but the strong invariance model exhibited substantial deterioration (M3 vs. M2: ΔCFI = −0.027, ΔRMSEA = 0.007). Releasing the intercept constraint for Item 8 (MI = 197.03, E.P.C. = 0.36) yielded a partial strong invariance model (M4) with acceptable changes in fit indices (ΔCFI = −0.009, ΔRMSEA = 0.002, relative to M2). These results support the comparability of latent constructs across T1 and T2, permitting subsequent cross-lagged panel analyses.

### 3.3. Model Comparison and Selection

In the CLPM analysis, four competing models were tested. Model 1 (M1: stability model) included only autoregressive paths and concurrent correlations among variables, without any cross-lagged paths. Model 2 (M2) extended M1 by adding a cross-lagged path from academic burnout to smartphone use addiction. Model 3 (M3) extended M1 by adding a cross-lagged path from smartphone use addiction to academic burnout. Model 4 (M4) included all autoregressive paths, concurrent correlations, and bidirectional cross-lagged paths. Chi-square difference tests indicated that M2, M3, and M4 all fit the data significantly better than M1, and that M4 provided a significantly better fit than both M2 and M3. Therefore, M4 was selected as the final model for subsequent analyses (see Table 3).

### 3.4. Cross-Lagged Panel Analysis of Academic Burnout and Smartphone Use Addiction

**Figure 1:**
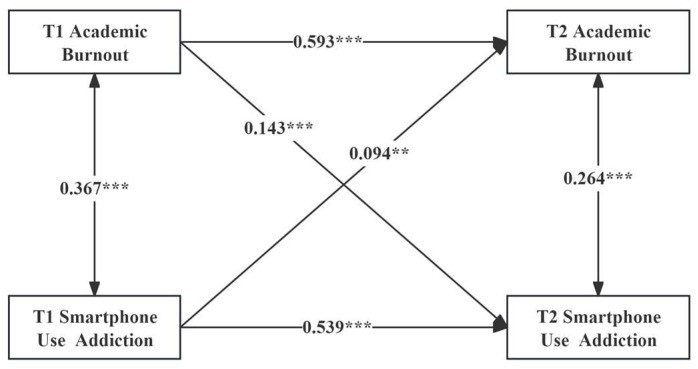
Cross-lagged Panel Model of Academic Burnout and Smartphone Use Addiction. Note: ** *p* < 0.01, *** *p* < 0.001.

The results of model comparison supported the adoption of Model 4 (the bidirectional CLPM). Controlling for gender, age, family residence, and income, we performed a cross-lagged panel analysis with T1 academic burnout and T1 smartphone use addiction specified as predictors and their T2 counterparts specified as outcomes (see Figure 1). The proposed model showed good fit to the data: *χ*^2^/*df* = 2.211, CFI = 0.990, TLI = 0.984, RMSEA = 0.037.

Results indicated moderate-to-high stability for both variables over the six-month period (academic burnout: β = 0.593, *p* < 0.001; smartphone use addiction: β = 0.539, *p* < 0.001). Cross-lagged analyses showed that T1 academic burnout significantly predicted T2 smartphone use addiction (β = 0.143, *p* < 0.001), and T1 smartphone use addiction likewise significantly predicted T2 academic burnout (β = 0.094, *p* < 0.01). By the empirical benchmarks established for cross-lagged panel models—where 0.03, 0.07, and 0.12 correspond to small, medium, and large effects, respectively (Orth et al., 2024)—the prospective effect of academic burnout on smartphone use addiction qualifies as a large effect, and the reverse path falls in the medium-to-large range. Thus, the bidirectional association between academic burnout and smartphone use addiction was supported, and Hypothesis 1 was supports.

### 3.5. FoMO as a Bidirectional Mediator in the Association Between Academic Burnout and Smartphone Use Addiction

**Figure 2:**
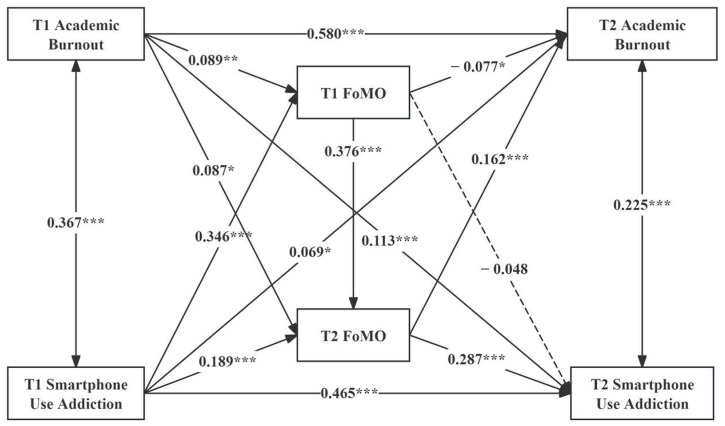
Cross-lagged Panel Model of Academic Burnout, FoMO and Smartphone Use Addiction. Note: * *p* < 0.05, ** *p* < 0.01, *** *p* < 0.001.

With demographic variables (gender, age, income, and family residence) included as covariates, cross-lagged panel models with mediating effects were specified, treating T1 academic burnout and T1 smartphone use addiction as predictors, FoMO at T1 and T2 as mediators, and T2 academic burnout and T2 smartphone use addiction as outcomes (see Figure 2). The model showed an adequate fit to the data, as evidenced by the following fit indices: *χ*^2^/*df* = 2.371, CFI = 0.989, TLI = 0.973, RMSEA = 0.039. Regarding antecedent paths, T1 academic burnout positively predicted T2 academic burnout (β = 0.580, *p* < 0.001), T2 smartphone use addiction (β = 0.113, *p* < 0.001), T1 FoMO (β = 0.089, *p* < 0.01) and T2 FoMO (β = 0.087, *p* < 0.05). Similarly, T1 smartphone use addiction positively predicted T1 FoMO (β = 0.346, *p* < 0.001), T2 FoMO (β = 0.189, *p* < 0.001), T2 academic burnout (β = 0.069, *p* < 0.05) and T2 smartphone use addiction (β = 0.465, *p* < 0.001). T1 FoMO predicted T2 FoMO (β = 0.376, *p* < 0.001). Notably, T1 FoMO significantly negatively predicted T2 academic burnout (β = −0.077, *p* < 0.05), but did not significantly predict T2 smartphone use addiction (β = −0.048, *p* > 0.05). These findings contradict theoretical expectations. In contrast, T2 FoMO positively predicted both T2 smartphone use addiction (β = 0.287, *p* < 0.001) and T2 academic burnout (β = 0.162, *p* < 0.001).

A bias-corrected percentile bootstrap method was used to examine the mediation effects (Wen & Ye, 2014). The 95% confidence intervals for five of the six indirect paths excluded zero, indicating significant mediation effects (see Table 4). Specifically, significant indirect effects were found through: (a) chain mediation involving both T1 and T2 FoMO sequentially (Paths 1 and 4); (b) simple mediation via T2 FoMO alone (Paths 3 and 6); and (c) simple mediation via T1 FoMO for the pathway from smartphone use addiction to academic burnout (Path 5), though notably this effect was negative (β = −0.027, 95% CI [−0.052, −0.003]). The indirect effect via T1 FoMO alone for the pathway from academic burnout to smartphone use addiction (Path 2) was not significant. These findings provide partial support for hypothesis H2, demonstrating that FoMO mediates the reciprocal association between these two variables, albeit with differing patterns across the two time points and directions.

## 4. Discussion

This study prospectively examined bidirectional associations between academic burnout and smartphone use addiction across two time points, while testing FoMO as a longitudinal mediating mechanism. Results suggested bidirectional effects between academic burnout and smartphone use addiction over the six-month period, alongside significant indirect effects via FoMO. Specifically, academic burnout predicted later smartphone use addiction through both chain mediation (operating through T1 FoMO to T2 FoMO) and single mediation (T2 FoMO only). Conversely, smartphone use addiction predicted later academic burnout through the chain pathway as well as single-stage mediation via either T1 or T2 FoMO. These results extend Davis’s (2001) Cognitive-Behavioral Model by demonstrating FoMO’s role in mediating reciprocal risk pathways between academic burnout and smartphone use addiction.

### 4.1. Bidirectional Longitudinal Associations Between Academic Burnout and Smartphone Use Addiction

Consistent with previous findings, this study indicates that academic burnout significantly predicts their smartphone use addiction six months later (Cheng & Wu, 2025; Tian et al., 2025; R. Wu et al., 2023; G.-H. Yang et al., 2024). According to Compensatory Internet Use Theory, when basic psychological needs are not fulfilled in offline life, individuals tend to seek compensatory satisfaction in virtual environments (Kardefelt-Winther, 2014). Such virtual gratification reinforces online engagement, which may eventually contribute to addictive patterns. Upon entering university, academic pursuit becomes the central developmental task for students and a critical criterion for evaluating their competence. When students experience academic burnout, they struggle to fulfill their basic psychological needs through academic activities. As a result, they often resort to digital media in an attempt to manage and regulate distressing emotional states such as burnout, depressive symptoms, and anxiety (Tian et al., 2025; Tu et al., 2023). Mobile phones, given their portability and multifunctionality, serve as the most accessible gateway to the internet. Accordingly, academic burnout may increase students’ susceptibility to problematic smartphone use. Additionally, according to Self-value Orientation Theory, individuals’ sense of self-worth is largely shaped by their behavioral performance and achievement levels (S. Jin et al., 2017). In Chinese culture, academic performance is commonly viewed as a key indicator of personal value and capability. When students perform poorly in academic tasks, their self-worth becomes vulnerable to erosion. Under such circumstances, smartphones may serve as an alternative means for individuals to reconstruct their self-worth within virtual environments. However, long-term reliance on these external compensatory mechanisms may contribute to smartphone use addiction.

These findings further indicate that smartphone use addiction prospectively predicted academic burnout six months later. Students who struggle with smartphone use addiction tend to allocate substantial attentional resources to online activities, often at the expense of their academic engagement. This pattern may undermine learning interest, weaken motivation, and impair academic performance (Hawi & Samaha, 2016). Over time, the cumulative effect of these factors can contribute to academic burnout. Previous research suggests that individuals with smartphone use addiction tend to develop automatic “stimulus-response” associations through repetitive use (L. Chen et al., 2018; Wiers & Stacy, 2006). These conditioned associations manifest as attentional bias toward phone-related cues that precede self-regulation failures in addicted populations (Dean et al., 2019; Rinck et al., 2018). Consequently, university students with smartphone use addiction are more easily distracted by notifications during learning, struggle to complete tasks on time, and face increased risk of academic decline, thereby contributing to academic burnout (Gao et al., 2021). Moreover, Media System Dependency Theory posits that as individuals’ reliance on a specific medium increases, the deleterious consequences of that medium become more severe (Ball-Rokeach & DeFleur, 1976). When university students rely excessively on smartphones over extended periods, their study time decreases markedly, leading to declining academic performance and diminished self-efficacy. Ultimately, this may result in maladaptive psychological outcomes, such as academic burnout (Junco & Cotten, 2011; Y. Wang et al., 2022).

### 4.2. The Mediating Role of FoMO Between These Two Variables

Results suggested that academic burnout longitudinally predicted higher smartphone use addiction, with FoMO mediating the association. This positions academic burnout as a distal factor in Davis’s (2001) Cognitive-Behavioral Model. Self-Determination Theory posits that effective self-regulation relies on the fulfillment of three fundamental psychological needs (Deci & Ryan, 2000). When these basic psychological needs remain unfulfilled, self-regulatory mechanisms are disrupted, which in turn increases individuals’ susceptibility to experiencing FoMO (Chai et al., 2018). Academic burnout represents a specific manifestation of unmet competence needs within the academic domain, reflecting significant deficiencies in psychological need satisfaction during learning activities. Students experiencing academic burnout often fail to derive a sense of accomplishment from offline academic pursuits. This deficiency may prompt them to seek compensatory gratification in virtual environments (Kardefelt-Winther, 2014). This compensatory pattern may foster excessive attention toward online information and generates irrational concerns about missing rewarding experiences (Elhai et al., 2020b). Consequently, to alleviate FoMO, individuals may engage in frequent smartphone checking behaviors, which reinforce psychological dependence on the device and may contribute to addictive patterns. Several studies have demonstrated that negative emotions resulting from unmet psychological needs, such as depression, are positively linked to FoMO (Oberst et al., 2017; Y. Wang et al., 2022), which subsequently predicts smartphone use addiction (Fioravanti et al., 2021). Taken together, these results support the mediating role of FoMO identified in the present study.

This research also suggests that smartphone use addiction significantly predicted academic burnout via FoMO, further corroborating FoMO’s bidirectional mediating role. Grounded in the displacement hypothesis (Kraut et al., 1998), excessive smartphone use may displace real-world social activities by consuming the time and psychological resources required for in-person interactions. Consequently, diminished offline interpersonal engagement may reduce opportunities for fulfilling social experiences, leading individuals to perceive themselves as excluded from rewarding real-world contexts and thereby contributing to elevated FoMO. These findings are consistent with previous related studies (Fang et al., 2026; Zhao, 2024). To date, FoMO has been predominantly viewed as a predictor of smartphone use addiction, with relatively few studies investigating whether smartphone use addiction reciprocally increases FoMO across time. Our findings suggest that T1 smartphone use addiction prospectively predicts T2 FoMO, a pattern potentially consistent with a reinforcement effect, though causal direction cannot be determined (Buglass et al., 2017). Notably, FoMO at different times exhibited divergent patterns in predicting T2 academic burnout depending on temporal proximity. Concurrently, T2 FoMO was positively associated with T2 academic burnout, indicating that these psychological states co-occur within the same assessment period—likely reflecting immediate resource depletion and attentional distraction. This research is consistent with previous related studies (C. Liu et al., 2022). Our findings further support Conservation of Resources Theory (Hobfoll, 2011). Conversely, after controlling for the baseline level of T1 academic burnout, T1 FoMO showed a weak negative prospective association with T2 academic burnout. Given the unexpected direction and small effect size of this association, we caution that this finding should be interpreted as exploratory rather than confirmatory. One post-hoc interpretation is that this weak negative association might reflect a transient compensatory pattern over time, though this remains speculative. Alternatively, this unexpected finding may be attributable to unmeasured confounding and fluctuation inherent in two-wave designs. Therefore, further research incorporating three or more waves is needed before any substantive theoretical or practical implications can be drawn.

### 4.3. Research Implications and Limitations

This research offers three primary contributions. First, this study extends Davis’s (2001) Cognitive-Behavioral Model by suggesting a reciprocal pattern of temporal associations between academic burnout and smartphone use addiction, with FoMO serving as a mediator. Second, the two-wave longitudinal design enables an examination of temporal associations among variables, thereby providing preliminary insights into the potential dynamic interplay between smartphone use addiction and academic adjustment. Third, these findings provide initial insights into potential mechanisms underlying this reciprocal associations, suggesting possible targets for intervention—specifically, interrupting the vicious cycle through FoMO management and enhanced satisfaction of basic psychological needs.

Although this research demonstrates several strengths, it also has certain limitations. First, the sample was drawn primarily from a single university in Guangdong Province. While representative of the local student population, its generalizability to other regions remains to be established through broader sampling. Second, the sample exhibited a gender imbalance, with 76.9% of participants being female. This skew is largely attributable to the recruitment of students from humanities and social science disciplines, where female students tend to be overrepresented in the Chinese higher education context. Consequently, this imbalance may limit the generalizability of our findings. Third, this study employed a two-wave longitudinal design, which, while allowing us to examine prospective associations and temporal order, cannot fully establish causality or capture non-linear developmental processes. Future studies with three or more time points are needed to test more complex mediational pathways and growth trajectories. Fourth, the traditional cross-lagged panel model (CLPM) employed in this study cannot disentangle stable between-person differences from within-person change. This limitation is particularly consequential given that the analysis was based on only two waves of data. Future research should therefore utilize three or more waves to permit random intercept cross-lagged panel modeling (RI-CLPM), thereby allowing trait-like stability and intra-individual dynamics to be parsed more cleanly.

## 5. Conclusions

Based on longitudinal evidence, this study draws two primary conclusions. First, these findings point to a pattern of bidirectional temporal associations between academic burnout and smartphone use addiction over the six-month interval. Specifically, each variable was temporally associated with changes in the other over time, suggesting a pattern of mutual influence rather than a unidirectional causal association. Second, FoMO significantly mediates this association, serving as a key cognitive-emotional pathway linking the two constructs.

## Figures and Tables

**Table 1:** Results of Descriptive Statistics and Correlations (N = 893).

	M	SD	1	2	3	4	5	6	7	8	9	10
1. Gender	—	—	1									
2. Age	19.93	1.24	−0.08 *	1								
3. Family Residence	—	—	0.01	−0.12 **	1							
4. Income	—	—	−0.01	−0.05	0.32 **	1						
5. T1 Academic Burnout	43.04	8.32	0.02	−0.03	−0.03	−0.04	1					
6. T1 FoMO	17.03	5.32	0.04	−0.10 **	0.03	0.06	0.22 **	1				
7. T1 Smartphone Use Addiction	43.85	10.21	0.11 **	−0.04	−0.04	0.02	0.37 **	0.38 **	1			
8. T2 Academic Burnout	41.45	8.16	−0.01	−0.10 **	−0.05	−0.05	0.63 **	0.15 **	0.31 **	1		
9. T2 FoMO	22.58	5.93	0.12 **	−0.08 *	0.05	0.07 *	0.24 **	0.47 **	0.37 **	0.29 **	1	
10. T2 Smartphone Use Addiction	49.22	11.37	0.16 **	−0.07	−0.06	0.03	0.34 **	0.29 **	0.60 **	0.42 **	0.47 **	1

Note: * *p* < 0.05, ** *p* < 0.01.

**Table 2:** Longitudinal Measurement Invariance Tests (N = 893).

Variables	Models	*χ* ^2^	df	CFI	TLI	RMSEA	SRMR	Comparison	ΔCFI	ΔRMSEA
Academic Burnout	M1: Configural	1109.27	431	0.924	0.912	0.042	0.058	—	—	—
	M2: Weak	1122.482	444	0.924	0.915	0.041	0.058	M2–M1	0	−0.001
	M3: Strong	1210.165	457	0.916	0.908	0.043	0.059	M3–M2	−0.008	0.002
Fear of Missing Out	M1: Configural	312.647	88	0.954	0.937	0.053	0.049	—	—	—
	M2: Weak	327.726	94	0.952	0.939	0.053	0.051	M2–M1	−0.002	0
	M3: Strong	363.941	100	0.946	0.935	0.054	0.053	M3–M2	−0.006	0.001
Smartphone Use Addiction	M1: Configural	1384.202	480	0.929	0.917	0.046	0.069	—	—	—
	M2: Weak	1460.84	493	0.924	0.913	0.047	0.073	M2–M1	−0.005	0.001
	M3: Strong	1805.675	506	0.897	0.886	0.054	0.077	M3–M2	−0.027	0.007
	M4: Partial Strong	1583.84	505	0.915	0.905	0.049	0.076	M4–M2	−0.009	0.002

Note. Based on modification indices (MI = 197.03, E.P.C. = 0.36), the intercept constraint for Item 8 was released in M4; changes were computed relative to M2.

**Table 3:** Results of Model Comparison (N = 893).

Model	Fit Index					Model Comparison			
	*χ* ^2^	df	CFI	TLI	RMSEA	Model	Δ*χ*^2^	Δdf	*p*
M1	57.89	10	0.951	0.936	0.073	—	—	—	—
M2	29.06	9	0.979	0.970	0.050	M2 vs. M1	28.83	1	<0.001
M3	42.90	9	0.965	0.950	0.065	M3 vs. M1	14.99	1	<0.001
M4	17.69	8	0.990	0.984	0.037	M4 vs. M1	40.20	2	<0.001
						M4 vs. M2	11.37	1	<0.001
						M4 vs. M3	25.21	1	<0.001

**Table 4:** Significance Test of Mediation Effects (N = 893).

Path		β	*SE*	*p*	95% Confidence Intervals
Path1	T1 Academic Burnout → T1 FoMO → T2 FoMO → T2 Smartphone Use Addiction	0.01	0.004	<0.05	[0.003, 0.018]
Path2	T1 Academic Burnout → T1 FoMO → T2 Smartphone Use Addiction	−0.004	0.003	>0.05	[−0.014, 0.001]
Path3	T1 Academic Burnout → T2 FoMO → T2 Smartphone Use Addiction	0.025	0.01	<0.05	[0.007, 0.048]
Path4	T1 Smartphone Use Addiction → T1 FoMO → T2 FoMO → T2 Academic Burnout	0.021	0.006	<0.001	[0.012, 0.034]
Path5	T1 Smartphone Use Addiction → T1 FoMO → T2 Academic Burnout	−0.027	0.012	<0.05	[−0.052, −0.003]
Path6	T1 Smartphone Use Addiction → T2 FoMO → T2 Academic Burnout	0.031	0.009	<0.01	[0.016, 0.052]

## Data Availability

Data is available upon reasonable request.
